# Editorial: Small ruminant breeding in the age of genomics

**DOI:** 10.3389/fgene.2022.1004445

**Published:** 2022-11-24

**Authors:** Tatiana Deniskova, Mario Barbato

**Affiliations:** ^1^ L.K. Ernst Federal Research Center for Animal Husbandry, Podolsk, Russia; ^2^ Department of Animal Science, Food and Nutrition, Università Cattolica del Sacro Cuore, Piacenza, Italy

**Keywords:** sheep, goats, SNP, genes, transcriptomes analysis

Since domestication, sheep and goats have provided humankind with food including meat and milk, along with wool and leather. Unlocking the genetic potential and promoting the further improvement of economically important traits is key to guaranteeing the sustainable management of sheep and goat populations in the coming decades. In this regard, the implementation of genomic tools facilitates the selection progress and provides valuable information to design educated conservation programs.

Goat genomics is a rapidly evolving field; however, there are several unexplored aspects in need to be unveiled to understand the genetic mechanisms underlying traits of practical interest (growth and development, meat, and wool production). Genes expressed in the pituitary gland are key regulators of animal growth processes. Possible differences in the expression patterns of relevant growth-related genes obtained from the pituitary gland of 36-week-old animals have been explored in goats raised under different management systems (Ncube et al.). *Growth Hormone 1 (GH1)* and *Insulin Like Growth Factor 1(IGF 1)* were found to be highly expressed in goats raised under intensive rather than extensive breeding systems.

The molecular mechanism involved with skeletal muscle differentiation was investigated in the Leizhou goats by Ye et al. as a base of information for increasing meat quality and quantity. Based on RNA sequencing, 991 differently expressed mRNAs and 39 differentially expressed miRNAs were found and were mainly enriched in calcium ion binding, ECM-receptor interaction, and focal adhesion. In addition, RNA sequencing analysis provided evidence that muscle differentiation takes place between 90 and 120 days of the fetus in Leizhou goats.

The effectiveness of the genomic selection depends on the applied DNA array density and optimal reference population size. Based on simulations, the medium marker density panel (45k SNPs) was found to ensure accuracy in estimating the genomic estimated breeding values (GEBV) and was recommended for genomic selection in goats (Yan et al.). A reference population size of 1,500 was proposed as optimal to achieve noticeable genetic progress in genomic selection for fiber diameter and live body weight in Cashmere goats. The accuracy of the GEBV was higher based on 100 QTLs for live body weight and on 50 QTLs for fiber diameter.

Male fertility including testis size is among the key factors of effective artificial insemination. Transcriptomic profiling of sheep testes at different developmental stages was explored based on the identification of differentially expressed genes (DEGs) by RNA-Seq (Xu et al.). Gene ontology analysis illustrated that detected DEGs were mainly involved in testis development and spermatogenesis. Kyoto Encyclopedia of Genes and Genomes (KEGG) analysis revealed the presence of several reproduction-associated DEGs such as *collagen type I alpha 1 chain* (*COL1A1*), collagen type I alpha 2 chain (*COL1A2*), *Platelet Derived Growth Factor Subunit A (PDGFA)*, and *IGF*)*.* Based on using weighted gene co-expression network analysis (WGCNA), Xu et al. reported hub genes positively associated with testis size and negatively associated with testis size. The first hub included *Ran binding protein 9* (*RANBP9*)*, Dynein Axonemal Heavy Chain 17* (*DNAH17*)*, Spermatogenesis Associated 4* (*SPATA4*)*, Calcium and Integrin Binding Family Member 4* (*CIB4*)*,* and *Spermatid Maturation 1* (*SPEM1*) genes. The second one comprised of CD81 Antigen (*CD81*), C-Terminal Src Kinase (*CSK*), *PDGFA*, Vimentin (*VIM*)*,* and *Inhibin Subunit Beta A* (*INHBA*).

The safety and health of lambs is the basis for cost-effective breeding. Gastrointestinal nematodes are parasites causing suffering in young animals and significant economic losses to sheep owners. The attempt to unlock the genetic variants associated with resistance or susceptibility to this parasite was performed based on linear regression and case-control genome-wide association studies in Katahdin sheep (Becker et al.). Case-control GWAS detected two significant SNPs (*p*-values 1.49e-08 to 1.01e-08) within introns of the gene *adhesion G protein-coupled receptor B3* (*ADGRB3*) associated with lower fecal egg counts. Linear regression analysis revealed four significant SNPs (*p*-values 7.82e-08 to 3.34e-08) located within the first intron of the gene *EGF-like repeats and discoidin domains 3* (*EDIL3*). Further, Notter et al. studied single nucleotide polymorphism effects on lamb fecal egg count (FEC) estimated breeding values in progeny-tested Katahdin sires. On chromosome 5 the largest SNP effects were at 63, 67, and 70 Mb, with LD among these SNP of *r* ^2^ ≤ 0.2. Chromosome-level regional heritability mapping indicated that one 500-SNP window between 65.9 and 69.9 Mb accounted for significant variation in PFEC EBV. This window included rarely identified markers for parasite resistance as the *interleukin 12B gene (IL12B)* at 68.5 Mb.

Signatures of positive selection in the sheep genome are usually detected on autosomes, while no such information is available for the X-chromosome. The search for adaption-related genes was performed to provide a map of the positive selection of the X-chromosome in a sample of eight native Croatian breeds representing the East Adriatic metapopulation and local mouflons (Mario Shihabi et al.). To achieve this goal, the authors implement a novel approach called Haplotype Richness Drop (HRiD) using only the information contained in male haplotypes. Besides three classical approaches as extreme Runs of Homozygosity islands, integrated Haplotype Score, and the number of Segregating Sites by Length were used as well. In total, 14 positive selection signals containing 34 annotated genes were identified. The most reliable selection signal was mapped by all four approaches in the region, overlapping between 13.17 and 13.60 Mb, and assigned to the CA5B, ZRSR2, AP1S2, and GRPR genes. The reported HRiD offers an interesting possibility to be used complementary to other approaches or when only males are genotyped, which is often the case in genomic breeding value estimations.

